# Diseases of the Period of Dentition

**Published:** 1885-12

**Authors:** W. C. Barrett

**Affiliations:** Buffalo, N. Y.


### Independent Practitioner.

VOL. VI. DECEMBER, 1885. No. 12.

### Original Communications.

### DISEASES OF THE PERIOD OF DENTITION.

AN ADDRESS DELIVERED BEFORE THE AMERICAN ACADEMY OF DENTAL SCIENCE AT ITS EIGHTEENTH ANNUAL MEETING HELD IN BOSTON, NOVEMBER 4, 1885.

BY DR. W. C. BARRETT, BUFFALO, N. Y.

Wordsworth truly said—"The child is father to the man,"—and the aphorism is pregnant with a great truth, for it is upon the impressible nature of childhood that the great influences are at work which shall mould, and shape, and determine the character of the young man. We all know how potent are the associations and influences of childhood, and how impossible it is for us, in after years, to divest ourselves of the impressions that may then have been stamped upon us. The habits and customs, the faiths, the beliefs, the superstitions which we may have acquired in manhood, may be laid aside like a worn garment. But the rules, the tenets, the habitudes, the prejudices, the very methods of thought which we acquire in childhood, hold dominion over us till the final end shall come.

It is emphatically true, then, that in the mental world the child *is* father to the man. Nor is it less the fact in the physical domain. As the twig is bent the tree is inclined. As this body of ours, composed of so many separate organs, is trained in infancy and youth, so will it grow up to manhood. Vicious habits of life, early acquired, will cling to us in old age. That intricate piece of machinery, the digestive apparatus, may easily be put out of repair in infancy.

Once fairly regulated and set in methodical motion, it is proof against the results of misuse. The delicate nervous organization, by irregular habits in youth, may become like sweet bells jangled, out of tune and harsh. But the period of youth once passed in perfect health, all the subsequent unnatural drafts of our busy lives will be duly honored.

Old Adam, in Shakespeare's "As You Like It," says:

> "Though I look old, yet I am strong and lusty;
> For in my youth I never did apply
> Hot and rebellious liquors in my blood;
> Nor did not with unbashful forehead woo
> The means of weakness and debility:
> Therefore my age is as a lusty winter,
> Frosty but kindly."

It is the irregularities of youth, and not the excesses of maturity, that strew the highways of life with so many wrecks of manhood.

If then, the human race is to be improved, if particular organs are to be rescued from the results of inheritance or of predisposition to disease, the work should be begun with existence, and hygienic measures must be adopted in early life. The teeth of the human race are the organs which soonest fail, while there are few upon which the comfort of age is more dependent. I could not, then, offer for your consideration at this time a subject of greater importance than that of the teeth during childhood. I shall not consider it from an anatomical or a histological stand-point, but shall confine myself to a physiological consideration of the matter.

The eruption of the deciduous teeth is not of itself a process that should be attended with any very serious disturbances. It is as purely physiological as is the growth of the hair or the nails, to which the teeth, as epidermoid structures, are allied, yet the period of dentition is almost universally recognized as the most perilous of our whole existence. That the dangers and the disturbances which are too frequently the accompaniments are due to the mere eruption of the teeth, I cannot believe. I am rather of the opinion that most of the febrile disturbances and the gastric irritations of the period of first dentition are coincidental, and due to other causes, and it is the object of this paper to examine some of these phenomena, and to trace them to their real cause.

The digestive apparatus undergoes great changes during existence. Not only is the histological structure of the organs different in the infant from those of the adult, but their functional action undergoes important changes. The character of the secretions is markedly different at different ages. Previous to birth, while the fœtus is deriving its nourishment from the blood of the mother through the placenta, the digestive apparatus is mainly dormant, and the different glands, which as yet have not been called into active exercise, provide no digestive and but little of other fluids. The lungs, too, are not as yet exercising their function, and the mother breathes, as she also provides nutriment, for both. But immediately upon the severance of the umbilical cord, or the stoppage of the placental circulation, the first stupendous change of conscious existence takes place.* The blood, no longer purified through the lungs of the mother, must have its separate and individual process for oxygenation, and this change, unlike the previous and subsequent modifications of growth, must be instant, and not gradual.

The circulation of the human embryo has already undergone one change *in utero*. Previous to the formation of the placenta, when the umbilical vesicle was the sole source of nutrition, there was a complete round of the circulatory fluid in the embryo tissues, and that circulation, like that of extra-uterine life, was wholly within itself. The vitellus was the organ which provided the fœtus with nourishment, and the vitelline circulation was that which carried pabulum to the formative tissues. But with the formation of the allantois the umbilical arteries came into existence. As the umbilical vesicle diminished, the allantois enlarged and was changed into a vascular chorion, and a part of this became the placenta which, during the greater part of intra-uterine life, supplied the growing fœtus and carried on its circulation.

These changes were gradual, but at the period of birth commences another circulation. The blood is now almost instantaneously directed to the lungs, which by the first feeble cries of the newly born

---

* It should be remembered that the first attempt at breathing is involuntary, and succeeds the stoppage of the supply of oxygen from other sources. If a pregnant animal be thoroughly anæsthetized, the uterus opened and the fœtus, it matters little at what period of existence, exposed to the air, there is no attempt at breathing so long as the placental circulation is kept up. But shortly after the severing of the umbilicus the tissues begin to feel the need of oxygen, and there are spasmodic attempts at breathing. If the fœtus be not sufficiently advanced for this it dies of suffocation. But if it can breathe, separate existence at once commences, by the establishment of the pulmonary circulation.

child are inflated with air, and so commences adult circulation. The ductus arteriosus, which, during fœtal life, had conveyed the blood directly from the right ventricle to the aorta, without its entering the pulmonary artery and the consequent pulmonary circulation, now closes up, while the pulmonary artery enlarges, and the blood is forced by the right ventricle through it, and thus independent oxygenation commences by the entrance of the lungs upon the active performance of the function of respiration.

All through these successive changes alterations are going on in the digestive organs. During fœtal life the alimentary canal has become partially filled with an accumulation which has received the name of meconium. The gastric juice is not yet secreted, although the liver provides a very scanty secretion. But at birth the digestive organs are called into active exercise, and provide secretions that are capable of acting only upon the very simple food which must at first be the sole regimen of the newly born child. The saliva is not as yet provided with the ptyaline which, later in life, gives it its distinctive character, nor are the gastric or intestinal juices yet of a nature that permits them to act upon any kind of solid food. Nature has provided the proper pabulum in the milk of the mother, which at first contains the colostrum that shall lead to the expulsion of the meconium which the intestines contain. With the development of the child the milk of the mother undergoes changes which gradually lead up to a stronger diet.

The transitions in the development of the digestive tract extend to full adult life. They are gradual, but they may be divided into successive and well marked stages. The development of the teeth keeps pace with them, and may, in all normal conditions, serve as an unfailing index of the state of advancement of the internal organs. At first there are no teeth, for the condition of the digestive tract is such that it cannot prepare for assimilation any form of solid food. The pabulum must be of the simple, mild character of the milk of the mother, which contains in solution all the nutrient forces needed by the immature organism, and in the exact proportion that nature demands. The substitution of this natural food by any form of artificial food is always more or less disastrous in its results. Nor can an infant of but a few days of age be safely given the milk of a woman whose confinement took place some time previous to the birth of the child. The milk of such a woman is too highly organized for its immature function. When young calves or lambs are deprived of the milk of the mother, and fed on that of others, who have been in lactation for some time, they do not thrive well. This is even more marked in the human race, because the period of lactation should be longer, and the changes are more gradual.

With the growth of the infant there is a progressive development of the teeth, the incisors appearing first. Too many mothers consider this the period at which the giving of solid food may commence. A greater mistake could not be made. For obvious reasons the teeth are successive in making their appearance, and the fact that the small deciduous molars, which alone are effective in mastication, do not appear until some months after the cutting of the incisors, should teach every mother that it is not until all the deciduous teeth are in position that the diet of the child may with any degree of safety be changed. Up to this time its exclusive food should be the milk of the mother. But with the appearance of the molar teeth the alteration in the digestive function becomes more marked.

It is about at this period that the character of the saliva undergoes a change. The great parotid gland now begins to pour through the duct of Steno, which is directly opposite the molar teeth, a fluid that has distinct parotid qualities. Did time allow, I should be glad to enlarge upon the peculiar properties of this parotid fluid, as distinguished from that of the other salivary glands. It must suffice to say that it is peculiarly fitted to so change the character of some foods as to be essential in their proper preparation for the digestive fluids, and that it is poured out in great quantities during the process of mastication in the precise place, where, if the molar teeth be present, it may be thoroughly mixed with the bolus, previous to deglutition.

The full development of the deciduous molar teeth, then, marks an important era in the growth of the digestive organs, and is an indication that they are sufficiently advanced to receive a diet of greater consistency. Succulent vegetables may now be digested, but the character and size of the teeth show that they are intended only for very light use, and that they, as well as the stomach and intestines, are not as yet fitted for the more highly organized animal foods.

With the continued growth of the general system of the child, the digestive tract progresses in efficiency, and the time approaches when it is prepared to assimilate pabulum of a yet more advanced character, and nature with careful hand is preparing the organs for the proper preparation of this food. Another and a larger molar makes its appearance—the largest tooth with which it is to be provided—and now for the first time the dental organs, and coincident with this the digestive organs, are sufficiently advanced to receive animal food of a simple character, and the more dense of the vegetable pabulums. As growth continues, the diminutive and feeble deciduous teeth are replaced by larger and stronger ones, until, when the age of about twelve years has been reached, the dental structure is complete, and the alimentary canal has reached a period of development that fits it for the digestion and proper preparation for assimilation of any food that is fit for alimentation.

The development of the whole body, if we except certain special organs, has thus advanced by regular gradations. Not entirely by imperceptible growth, for, as in inanimate nature, there are periods of pushing energy, and others of comparative rest. It is by alternation of rest and effort that all organic changes occur. But the general advance of the system is along the same line, and in studying the development of any one organ, or set of organs, it is necessary to consider their relation to the growth of other organs. "To regard the eruption of the teeth as the sole factor in the general process known as the first dentition, is to perpetrate a kind of medical synecdoche. Children get their first teeth because they are at the same time getting a second stomach and second intestines." *

It may thus be seen that the condition and growth of the internal organs of digestion may be accurately gauged, and their capabilities for work exactly determined, by the state of dental development. If, previous to the appearance of the teeth fitted for its perfect mastication, any special food be given the child, it cannot be digested, but must act as an irritant, provoking all kinds of gastric and febrile disturbances, thus not only delaying the progressive development of the alimentary organs, but actually causing their deterioration and the destruction of their functional powers.

To my apprehension it is from the lack of the knowledge of this developmental progression, or from willful disregard of the lessons

* Pediatric Aphorisms. Prof. Letamendi. INDEPENDENT PRACTITIONER, October, 1884, page 591.

which it so plainly teaches, that the most of the so-called diseases incident to teething arise. As I have said, the growth and eruption of the teeth is a physiological process. If they be accompanied by any pathological conditions, these are due to extraneous and ordinarily preventable causes. It is to errors of diet that they should usually be ascribed, and not to the mere advancement of normal organs. An excellent authority says: "While not denying that certain minor evils may attend the evolution of the teeth, it is doubtful if many of the more serious ailments, often accompanying this process, have any dual connection with it." *

Another writer upon the same subject says: "Dentition is usually held to be the cause of many ailments, but to what extent it is really so is doubtful. The time of dentition is one of transition. A uniform and bland diet is changing for one of greater variety, and the febrile attacks, diarrhœa and vomiting, which are so rife at this time, are more satisfactorily explained by indigestibility of food than by some occult influence of tooth-cutting."†

Dr. Edward Henoch, of Berlin, says: "In the opinion of a majority of physicians teething is a physiological process, that cannot give rise to any morbid phenomena." ‡

Another competent authority says: "No doubt during the cutting of the teeth, the bowels generally are in a state of irritability, for we know that at these periods the follicular apparatus of the intestines is undergoing considerable development." §

The so-called diseases incident to teething are mainly gastric disturbances. The period of greatest fatality corresponds very nearly with the time of the eruption of the teeth, because it is about this time that most mothers commence a change of diet for the child. With the appearance of the incisors solid food is given. The infant, with the instincts of nature, rejects it, but the mother persists until a taste for unfit food is acquired, very much as an appetite for tobacco is perhaps obtained in later years. At first, the amount ingested is insufficient to produce serious consequences, and as larger amounts are taken a diarrhœa of no alarming kind manifests itself. The appetite for unfit solid food increases with its gratifica-

---

* Archives of Pediatrics; June, 1885, page 380.

† Goodhart. Diseases of Children. Page 28.

‡ Edwards. Therapeutics of Diseases of Children. Page 105.

§ Eustace Smith. Wasting Diseases of Infants and Children. Page 67.

tion, and soon febrile symptoms supervene. The child becomes exceedingly restless. The milk which it takes is regurgitated—perhaps there is vomiting of some curdled milk—the diarrhœa increases, and the discharges have an excoriating effect, until finally digestion stops and the child dies of inanition, or perhaps convulsions ensue, one of which proves fatal. And yet it will be said that the child died from teething, when dentition was progressing naturally, the gums were not swollen or tender to the touch, and there was no indication of any reflex nervous irritation.

Do not understand me as insisting that delayed dentition may not induce a train of symptoms somewhat analogous to these, but when fever and a slight diarrhœa are due to this cause, the diagnosis should not be difficult. In this case the disturbance will not be functional, but of a nervous character, and the gums will give timely indication and warning of the impending trouble. In by far the greater proportion of the diarrhœas, and the gastric forms of the diseases of young children, I am confident that a careful examination of all the attending circumstances will find that they are due solely to errors of diet. In my own clinics at the Medical Department of the University of Buffalo, I have scarcely met a case in which it was necessary to give the advancing teeth any alleviation whatever. Children have been presented for lancing of the gums, by mothers who were confident that all the little one's ailments were due to the cutting of its teeth. The family physician had perhaps told them so, but an examination of the mouth almost invariably found the gums in a healthy condition, and the teeth progressing normally. No child was ever yet presented at my clinic whose food was exclusively the milk of a healthy mother. But I have frequently seen them when their stomachs were distended by the products of the indigestion of food that was lying, an absolute foreign substance, within them. Consider, gentlemen, what is your own condition when suffering from the eating of improper food, and then think what must be the state of a little helpless infant, with all its susceptibility to malign influences. Its stomach is loaded with that which cannot be digested. The presence of the mass is intolerable to the organism, and it must be eliminated in some way. Nature hangs out the signal of distress in the form of a rise of temperature, and a general febrile condition, if it be not vomited up. The mass acts as an irritant, producing a violent motion of the coats of the stomach, and it is perhaps passed through the pylorus into the intestines, where it produces increased peristaltic action, and so is hurried through, leaving the whole digestive tract in such an inflamed and angry condition that a diarrhœa of some days' existence is the natural sequence. This is repeated time and again, until a chronic condition of irritation ensues and the child dies of the exhaustion consequent upon it. Or, perhaps, the undigested food remains in the stomach, the powers of the child having been so exhausted by previous struggles that the mass cannot be eliminated. In this case convulsions will probably end the scene, and if one or two teeth be, as they usually are at this period of life, just making their appearance through healthy gums, they will be charged with the untimely death.

A thoughtful writer in one of our journals says. "I do not ignore the potent agency of the various meteorological changes in the causation of these fatal diseases in the infant, but I am convinced that many more children would be saved during such changes if more attention were paid to alimentation as a prophylactic measure. Experience has taught me that in those families where I was allowed to regulate the food of the infant, and where there was no departure from the rule laid down, the children passed through a very hot summer without being subjected to the ailments which are so disastrous to early life. If, however, parents are permitted to use their own judgment, or permit themselves to be influenced by the whims of some motherly neighbor, who scoffs at the present scientific dietary and boasts that she has reared a family of twelve children, and fed them 'from the table' when they were babies, then the physician might as well pursue the 'let alone plan' with almost a guarantee that his services will soon be required to pacify nature offended by a supper of bacon and cabbage." *

I have said that delayed dentition may produce symptoms that closely simulate those consequent upon improper feeding. How shall we distinguish between the two? Parents will consult us upon the advisability of lancing the baby's gums as a cure for its restlessness, pain and diarrhœa. It is important that we be able to set the family physician right, if he be wrong, and to determine with certainty whether there exists a necessity for the dentist's services. It

* S. S. Adams, M. D. "How Shall We Feed the Baby." Archives of Pediatrics, May, 1885, page 270.

is as essential that we know what to avoid, as to tell what to do. If we can demonstrate to the anxious mother that we fully comprehend the situation, we shall gain her respect and confidence, and she will be likely to heed our admonitions regarding other dental troubles.

A correct diagnosis can only be made with certainty after a very careful examination, not only of the child itself and the attending symptoms, but of its past history, its sanitary environments, and its diet. The age should be accurately determined, that it may be seen whether the dental development corresponds with that of the general system. This is important, because it is not infrequent that morbid conditions are ascribed to teething when the teeth due at the time are all in place. A medical journal reports a case of infantile palsy in a child more than three years of age, as due to teething. Both legs were cold and powerless. There was sufficient irritation of the gastrocnemius muscles to cause a permanent contraction, thus producing a kind of talipes equinus.* Nothing is said about the state of forwardness of the dentition, but unless it was unusually delayed, the physician in this case, as is too often done, jumped at his conclusions, and ascribed to teething a trouble that had a deeper origin.

The condition of the gums should be carefully noted. If they are normal, without any special inflammation or thickening, and if on the other hand they be not unduly tense and glistening, we should look elsewhere for the source of the irritation. It should be remembered that the gum is naturally very hard and dense, from the large amount of fibrous tissue in it. Normal growth, when the tooth is near the point of emergence, will find the gum whitish, glistening, and tense in appearance. There may be such a condition of impermeability, of toughness and hardness in the gum, that the advancing tooth is retarded thereby, and hence undue pressure is brought to bear upon the, as yet, insufficiently protected pulp, thus inducing reflex nervous disturbances, but I believe this condition to be comparatively rare. Unless there be constitutional and general disturbances that seriously interfere and require immediate attention, the tooth easily makes its way through the gums, by their absorption under the slight but continual pressure of the growing tooth. Should the contrary be the case, the remedy is a simple one;

* Medical Bulletin, June, 1885. Page 196.

a crucial incision with the lancet over the point of the tooth, which will be easily detected, will give it egress, and the operation will be a painless one.

The general symptoms attendant upon this unusual condition will be of a nervous character. The child will have periods of irritability, and they will be paroxysmal in character. It will suddenly awake from a sound sleep with a frightened cry. There will be spasmodic twitchings of the muscles, more especially of the face. It may give indications of earache. Its eyes will probably be watery, or possibly gummy. It will desire to bite upon hard substances, which will give it pain and cause it to cry out. Later on, however, this will change, and it will fear the contact even of the finger with the gums. There may be an increased or a diminished flow of saliva, or it may at one time be streaming from the mouth, and at another all the oral tissues may be parched and dry. All these symptoms will be, as before stated, paroxysmal in their nature, and it is this peculiarity which especially marks the condition. The bowels may be very irregular, or they may not be particularly affected. Sudden watery discharges may be succeeded by constipation. Their condition offers no very clear indication of the real cause of the trouble, for they may be in a comparatively normal state, unless there be gastric disturbances due to other causes.

A simple lancing of the gums over the advancing tooth, with the administration of a mild anodyne, will be all that is necessary. A four per cent. solution of the muriate of cocaine may be rubbed upon the gums, and this will afford a present relief, and by its action upon the terminal filaments of the nerves will frequently stop the muscular twitching, if this be present. Or the following lotion may be prepared and the gums bathed with it:

℞

| | | |
|---|---|---|
| Hydrochlorate of Cocaine, | grs. | iss. |
| Tinct Saffron, - | gt. | x. |
| Syrup Simp., - - | ℥ | iiss. |

I would advise a sparing use of the lancet if it be necessary to resort to it at all. The incisions need not be extensive, and they cannot be very deep, nor should they include the gum over teeth that are not manifestly retarded by actual pressure.

There is another and yet rarer condition of the oral tissues of teething children, that is of a more formidable character. It is when the teeth are slow in their development, when they are retarded and roughened through constitutional or general derangements, and thus become a long continued source of irritation. The gums are then turgid, inflamed, thickened, abnormally vascular and tender to the touch. It will be remembered that in a healthy state they are the direct opposite of this; that they have but a limited blood supply, few nerves, have comparatively little sensation, and are hard, smooth, light in color and glistening in appearance. The abnormal condition to which I refer is easily recognizable at a glance. The salivary secretion is largely increased. The inflammation and redness extends into the pharynx, and finally down the alimentary canal to the stomach, producing gastritis, and an irritating, exhaustive diarrhœa. If the sanitary conditions be bad and the food of an improper character or insufficient in nutritive power, the disease assumes the type of a malignant stomatitis, or even of cancrum oris. The tissue of the gums becomes ulcerative, and sloughing ensues. The child becomes excessively debilitated through its inability to take nourishment, and from the diarrhœa which accompanies the condition there is a general malaise. The mouth is hot, the tongue coated, the breath offensive, the urine is high in color and scanty, and all the tissues of the body sympathize in the disturbance.

That these symptoms may possibly be induced by delayed and vicious dentition, assisted and aggravated by bad sanitary conditions, I believe to be indisputable. I have seen some of these in a boy of fourteen years of age, brought about by retarded eruption of the cuspids, for it is not alone the cutting of the deciduous teeth that may induce serious disorders. Henoch says that he "considers dentition, both first and second, to have much to do with their occurrence." *

The first remedial measures in these diseases should be directed toward the general system. Bad sanitary conditions should be remedied and the strictest hygienic treatment instituted. The diet should be carefully regulated, and a moderate cathartic, followed by pepsin and dilute muriatic acid be given, if there be great digestive disturbances. If the symptoms are mainly oral, chlorate of potassium and muriatic acid should be given. The following local mouth wash may be used every hour :

* Goodhart. Diseases of Children. Page 88.

Potass, Chlorate, gr. x.
Aqua Dist., f℥ i.

I have frequently, in analogous conditions, seen the happiest results from the use of Listerine one part, water ten to twenty parts. If there be ulceration, the surfaces of the ulcers should be swabbed with a saturated solution of permanganate of potassium.

When the condition of the mouth and the general system will warrant it, surgical interference may be demanded. A careful examination, with due consideration of the age and state of development of the other teeth, will enable the judicious surgeon to determine what tooth or teeth may be delayed and acting as the irritant. Under the influence of an anæsthetic, if necessary, or five minutes after painting the gum over with the fluid extract of Cannabis Indica, deep incisions should be made over the point of the delayed tooth, and the gum dissected back a little until its exact condition can be determined. If it be bound down by adhesions, these should be cut. If the tooth be presenting abnormally, or if it be so impacted that its development will only aggravate the existing difficulty, it may be necessary to extract it, but these are conditions that it is not my present purpose to discuss. Impacted teeth usually bring about a train of symptoms quite distinct from those enumerated, and they require radical surgical interference. I am not speaking of anatomical anomalies, but of those pathological conditions which may accompany the eruption of normal teeth. It will usually be sufficient, in the inflammatory condition considered in this paper, to make deep and free incisions down to the offending tooth or teeth, and to cut or bur away any roughness of the surface that may be causing the irritation. I had occasion not long since to thus smooth the surfaces of two first permanent molars, whose enamel upon the crown was almost like a coarse wood rasp.

I have spoken of retarded dentition. This may occur because of a general lack of development, the teeth simply keeping pace with the growth of the rest of the body. There are but few constitutional diatheses which interfere with dentition. In inherited syphilis and in tuberculosis, the teeth are usually cut early. In chronic diarrhœa and in the weakness caused by improper food, the development of the teeth goes on uninterruptedly. There is but one disease in which general nutrition is affected, that seriously interferes with tooth growth, and that is Rachitis. Whenever the child is attacked by this, then development of the teeth is arrested. Eustace Smith says that this influence is peculiar to rickets. "If the disease makes its appearance before any of the teeth are cut, their evolutions may be almost indefinitely postponed. If some teeth have already appeared, the further progress of dentition is interrupted."*

It may not be unprofitable to glance at the symptoms attendant upon an improper diet, and which are in my opinion the usual cause of the so-called diseases incident to teething.

Diarrhœa is the most common of infantile diseases,and it is usually one of the first symptoms of a deranged nutrition. There are three elements which tend to produce this condition :

1st—The nervous reflex of dentition.

2d—Improper feeding.

3d—The unusual heat and moisture of the particular time of the year in which these attacks usually occur.†

I believe that the first of these is much less often a cause than is usually imagined, but it cannot be altogether excluded. The third undoubtedly has its influence, but by far the greater number of cases of diarrhœa in children may be directly traced to errors in diet. At the age of eight or nine months, when the incisor teeth are making their appearance, most mothers and nurses commence the giving of more solid foods. The period is one of unusual peril, for it is one of transition. There are eras during which unusual progress is made, and the time of dentition is one of them. The young child is then peculiarly subject to disorders of many kinds; not as a consequence of the eruption of the teeth, but as a part of a general activity of growth and development, to which dentition and morbid phenomena both in a sense respond.‡

At this critical period new articles of food, which the undeveloped nutrient organs are powerless to digest, are introduced into the stomach, and a portion passed into the intestines. Within a few hours the symptoms of indigestion are manifested. Perhaps there may be nothing more formidable than flatulency and colic of a mild character. Or there may be vomiting and purging for some days, with but little of febrile disturbances. If the bowels be not

* Wasting Diseases of Infants and Children. Page 97.

† The Summer Ailments of Teething Children. Judson Bradley, M. D. Detroit *Lancet*, October, 1885. Page 153.

‡ Goodhart. Diseases of Children. Page 29.

relieved the child becomes pallid, and is fretful and restless. A slight rise of temperature may be observed, and it is thirsty and will drink large quantities of water. The mouth is dry, the tongue red and furred, and its papillæ prominent. The evacuations become more frequent; they are liquid and perhaps green and offensive. Then the temperature suddenly jumps to 101° or 103°, and the evacuations become colorless, profuse and watery, with a sickening odor, or perhaps they assume a pinkish color. These symptoms may be succeeded by a sudden collapse, the temperature falling rapidly, and death ensue at but a few hours' notice. All these are the possible attendant symptoms upon a simple diarrhœa, the result of injudicious feeding, or they may follow as a train succeeding it. It will be seen that they vary, in important particulars, from those given as the results of reflex nervous irritation arising from the presence of an unrelieved, advancing tooth, and from those attending the inflammatory state of the gums due to roughened, unerupted teeth. It is important that the varying conditions be kept clear in the mind, that remedial measures may be properly directed.

Cholera infantum has been by some authors classed as one of the diseases due to teething. It would seem that a little reflection should show the fallacy of this theory. It is not necessarily confined to the teething period, nor are the symptoms such as would lead one to infer dental reflex nervous irritation. A debilitated system, unhealthy sanitary surroundings, and an existing, or even a recent, gastro-intestinal irritation, are the contributing or predisposing causes, while a continued high atmospheric heat, or sudden changes in a moderately oppressive temperature, and sudden changes in the diet, are usually the exciting causes.*

A healthy infant is suddenly seized with profuse purging and vomiting. The skin becomes moist and cold, the features assume a leaden pallor, the eyes are sunken, the fontanelles depressed, and the symptoms of an Asiatic choleraic collapse are present. It is from its resemblance to this latter disease that it derives the name of cholera infantum.

Malarial fevers are sometimes mistaken by the unthinking attendant for disturbances of teething. There will be vomiting and pain

*Cholera Infantum. William Perry Watson, M. D. Archives of Pediatrics, August, 1885. Page 451.

in the epigastrium, indigestion, with nausea and diarrhœa, sore throat arising from pharyngitis or tonsillitis, coryza, and some febrile symptoms. These indications need not, however, be mistaken for true cases of difficult dentition. It should be remembered that, in infantile malaria, the chill and sweating stages are always absent.*

Stomatitis, thrush, and cancrum oris are diseases of the oral cavity that have no direct connection with dentition, though a condition simulating them may rarely be seen as the result of ragged, roughened, retarded teeth. Acute stomatitis is usually distinguished by a very offensive breath, and the spitting of bloody saliva. The pillow at morning will frequently be found stained with blood. It usually takes the form of a superficial ulceration of the edges of the gums, tongue and buccal surfaces, the gums being very vascular and covered with yellowish, deteriorated granulations. Sometimes it appears as widely distributed apthous ulcers.

Thrush is a fungoid growth, which may be found upon the mucus membrane of the mouth. This fungus has received the technical name of *Oidium Albicans.*

Cancrum oris appears as a hard, indurated swelling in the cheek. This rapidly degenerates, extends and becomes gangrenous, eating its way through the soft tissues and destroying all within its reach. These last three conditions are usually found in ill nourished children, with bad sanitary surroundings, and a lack of hygienic precautions.

There is one other disease to which I will direct your attention, and which in certain circumstances may be mistaken for dental disturbances. I refer to infantile convulsions. These frequently simulate the epileptic convulsions of older people. The child will scream or cry violently before the fit, when it becomes unconscious, with tetanic spasms. Sometimes there is a tremor in the sleep, twitching of the lips, sudden startings, the thumbs or toes rigidly flexed, with convulsive movements of the eyelids. These may resemble the reflex nervous spasms sometimes caused by tooth pressure, but the nice observer will be enabled to distinguish them through exclusion, and by noting whether they be general in character, or confined more especially to the connections of the trigemini.

Experience and careful examination will enable one to distinguish

* Malaria in Children. J. P. Kingsley, M. D. *Courier of Medicine*, August, 1885. Page 103.

the comparatively few instances of general disturbances caused by dental irritation. You have already learned that, in my own opinion, these cases are rare. "The child is teething," is the vague explanation given to many an anxious mother, by practitioners who are either incompetent to form a complete diagnosis, or too indolent and careless to seek for the hidden springs of disease; who are too heedless to carefully weigh all the symptoms, masked as well as developed, and who trust to luck for a verification of their hastily formed prognosis. I believe that this convenient make-shift has quieted the alarmed mothers and lulled the anxious friends of countless little innocents into a fancied security, and stayed the fears which might otherwise have instituted timely measures for relief, until, too late to save the little one, it was discovered that an alarming disease had attacked the vital organs, the very citadel of life. "Only teething." To how many promising young existences in which were centered the hopes, the ambitions, the heart affections of a family circle, have these words sounded the knell. "Only teething,"—and the fond parents looked with but little alarm upon symptoms of the gravest character. "Only teething"—but when to their agonized ears came the sound of the clods of earth falling upon the little coffin, and they realized that with the words, earth to earth, dust to dust, ashes to ashes, the door which opened to a bright and promising future of years of comfort and happiness to be spent with the loved child when grown to manhood or womanhood was now forever closed, when all the world seemed but one wide churchyard in which to bury from their sight their loved ones, in this hour of anguish would it alleviate their pain to know that a great mistake had been made—that a fatal error had been committed? And if, to the conscientious physician, the knowledge too late should come that, to save himself a moment's labor and thought, he had lightly passed over indications that it was his duty faithfully to study, would not the sight of those grieving parents, the habiliments of mourning, "the knell, the pall, the groan, the tear," would not these things rest heavily upon his soul? That you, gentlemen, may be spared such vain regrets, the lashings of tortured conscience consequent upon manifest duty fatally neglected, I have endeavored, so far as my poor ability permits, to point out to you the danger of mistaking grave pathological conditions for mere functional derangements.

